# *Aspergillus fumigatus* Stimulates the NLRP3 Inflammasome through a Pathway Requiring ROS Production and the Syk Tyrosine Kinase

**DOI:** 10.1371/journal.pone.0010008

**Published:** 2010-04-02

**Authors:** Najwane Saïd-Sadier, Eduardo Padilla, Gordon Langsley, David M. Ojcius

**Affiliations:** 1 Health Sciences Research Institute and School of Natural Sciences, University of California Merced, Merced, California, United States of America; 2 Graduate Group “Biochimie, Biothérapies, Biologie Moléculaire et Infectiologie”, Université Paris Diderot, Paris, France; 3 Institut Cochin, Université Paris Descartes, CNRS UMR 8104, INSERM U1016, Paris, France; New York University, United States of America

## Abstract

Invasive aspergillosis (IA) is a life-threatening disease that occurs in immunodepressed patients when infected with *Aspergillus fumigatus*. This fungus is the second most-common causative agent of fungal disease after *Candida albicans*. Nevertheless, much remains to be learned about the mechanisms by which *A. fulmigatus* activates the innate immune system. We investigated the inflammatory response to conidia and hyphae of *A. fumigatus* and specifically, their capacity to trigger activation of an inflammasome. Our results show that in contrast to conidia, hyphal fragments induce NLRP3 inflammasome assembly, caspase-1 activation and IL-1β release from a human monocyte cell line. The ability of *Aspergillus* hyphae to activate the NLRP3 inflammasome in the monocytes requires K^+^ efflux and ROS production. In addition, our data show that NLRP3 inflammasome activation as well as pro-IL-1β expression relies on the Syk tyrosine kinase, which is downstream from the pathogen recognition receptor Dectin-1, reinforcing the importance of Dectin-1 in the innate immune response against fungal infection. Furthermore, we show that treatment of monocytes with corticosteroids inhibits transcription of the gene encoding IL-1β. Thus, our data demonstrate that the innate immune response against *A. fumigatus* infection involves a two step activation process, with a first signal promoting expression and synthesis of pro-IL-1β; and a second signal, involving Syk-induced activation of the NLRP3 inflammasome and caspase-1, allowing processing and secretion of the mature cytokine.

## Introduction

Invasive aspergillosis (IA) is a life-threatening disease that occurs in patients with hematological malignancies [1], [2], solid organ transplants [3], or immunodeficiency syndromes or patients receiving immunosuppressive treatment [4], [5]. The genus *Aspergillus* includes about 200 species, of which 20 have been reported as human pathogens causing opportunistic infections, allergic states and invasive aspergillosis.

*Aspergillus fumigatus* is considered as the second most-common causative agent of fungal infection after *Candida albicans*. *A. fumigatus* grows at physiological temperature (37°C), has a stable haploid genome, and undergoes asexual reproduction, forming conidiospores that are released into the environment. Due to their small size (2–3 µm in diameter), the conidia can penetrate deeply into the respiratory airway by simple inhalation and adhere to epithelial cells before infection starts [6]–[8].

Normally, this fungus is efficiently eliminated by the immune system in healthy individuals; however it can trigger a severe IA responsible for high rates of morbidity and mortality in immunocompromised people [9], [10]. In these patients, *Aspergillus* spores begin to germinate in the lungs, forming branching hyphal filaments that break off and enter the bloodstream, leading to vascular invasion throughout the body [11]. Almost all organs can be infected after fungal dissemination. Co-infection with other pathogens such as cytomegalovirus (CMV) or *Candida* is very common and complicates IA, making it harder to cure.

The innate immune response against *A. fumigatus* plays a crucial role in controlling infection [12]. Several pattern recognition receptors (PRRs) such as Toll-like receptor (TLR)-2, TLR-4 and dectin-1 [13] have been observed to play a role in recognition and clearance of the fungus [14]–[17]. These studies have shown that host resistance to *A. fumigatus* involves the induction of pro-inflammatory cytokines including INFγ, interleukin (IL)-12, TNFα, and significantly, IL-1β [18], [19]. Nevertheless, the immunostimulatory molecule(s) of *A. fumigatus* that are recognized by PRRs and the molecular basis for inflammation initiation are still under investigation.

PRRs sensors of conserved motifs expressed on microbial pathogens called “pathogen-associated molecular patterns” (PAMPs) [20]. PAMPs stimulate PRRs such as surface-bound and endosomal TLRs, but also dectin-1 and cytosolic NOD-like receptor (NLR) family members. Stimulation of these PRRs (TLR-2, TLR-4, and dectin-1) during infection with A. *fumigatus* subsequently leads to activation of transcription factors such as NF-κB, whose translocation into the nucleus stimulates the upregulation of pro-inflammatory cytokines. Secretion of pro-inflammatory cytokines (TNFα, IL-12 and IL-1β) and chemokines (Mip-2 in mice, IL-8 in humans) helps to recruit neutrophils and lymphocytes to the pulmonary infection site and insure clearance of the fungus. Neutrophils and macrophages are the two main cell types responsible for the innate host response against aspergillosis, therefore the risk of infection is higher in subjects presenting an inadequate number or anomalies of these cell types [21].

The pro-inflammatory cytokine, IL-1β, is synthesized as an inactive cytoplasmic precursor, pro-IL-1β, which is processed into a biologically active, secreted form by caspase- 1, a cysteine protease [22], [23]. The latter is synthesized as an inactive form that is self-activated by cleavage, generating an enzymatically active heterodimer composed of 10 and 20 kDa chains [22]. Recent studies have implicated members of the NLR family of proteins in the regulation of caspase-1 activation [22], [24]. The NLR family is composed of 23 cytosolic proteins, some of which recognize PAMPs. The family includes nucleotide binding oligomerization domain 1 (NOD1), NOD2 [12], [16], the NLRP3/cryopyrin/Nalp3 “inflammasome” component [25], [26], and the NLRC4/Ipaf inflammasome component [27], [28].

Upon infection, stimulation of TLRs or the cytosolic NOD1 or NOD2 receptors activates transcription, synthesis, and secretion of pro-inflammatory cytokines such as INFγ, IL-12, and TNFα [29], [30]. Given the key role played by IL-1β in fever and inflammatory disease [31], its production and secretion is tightly controlled and requires typically two separate signals [32]–[34]. The first signal comes from PAMPs and promotes transcription, production and intracellular accumulation of the immature cytokine. The second signal, usually derived from a “danger signal” (DS), leads to the activation of an inflammasome, activation of caspase-1, and secretion of the mature cytokine. The requirement for two signals thus insures that IL-1β is secreted by macrophages only if they are stimulated by PAMPs and the PAMPs are produced under circumstances that could be viewed as potentially dangerous to the host organism [25]. Examples of DSs include host-cell components released from dying, infected or stressed cells such as ATP, adenosine, uric acid, or chromosomal proteins; but they could also be microbial PAMPs that are located in “threatening” locations, such as flagellin in the cytosol of an infected cell [33].

Several studies have recently described stimulation of the NLRP3 inflammasome in the innate immune response to *C. albicans* infection [35]–[37]. These were the first reports to show the involvement of an inflammasome during a fungal infection. However, stimulation of an inflammasome has not been described yet during *A. fulmigatus* infection. Although caspase-1 activation during *A. fumigatus* infection has not been investigated, studies showing secretion of IL-1β by the human monocyte/macrophage cell line, THP-1, following stimulation by *A. fumigatus* [19] suggested that caspase-1 must be activated in these cells, either directly by the fungal pathogen or in combination with a host-cell derived DS.

The goal of this study was therefore to determine whether *A. fumigatus* induces IL-1β secretion in a caspase-1 dependent manner by THP-1 cells, and evaluate whether this fungus activates an inflammasome. Our results show that *A. fumigatus* spores fail to induce caspase-1 activation, unlike hyphal fragments, which upregulate pro-IL-1β synthesis and stimulate caspase-1 activation. Importantly we revealed the requirement of an NLRP3 inflammasome and its adaptor protein, apoptosis-associated speck-like protein containing a caspase recruitment domain (ASC), in activating caspase-1, thus revealing NLRP3 and ASC as key regulators of inflammation during *A. fumigatus* infection.

## Results

### *A. fumigatus* hyphae upregulate pro-IL-1β expression and induce IL-1β secretion in human monocytes

**Figure 1:**
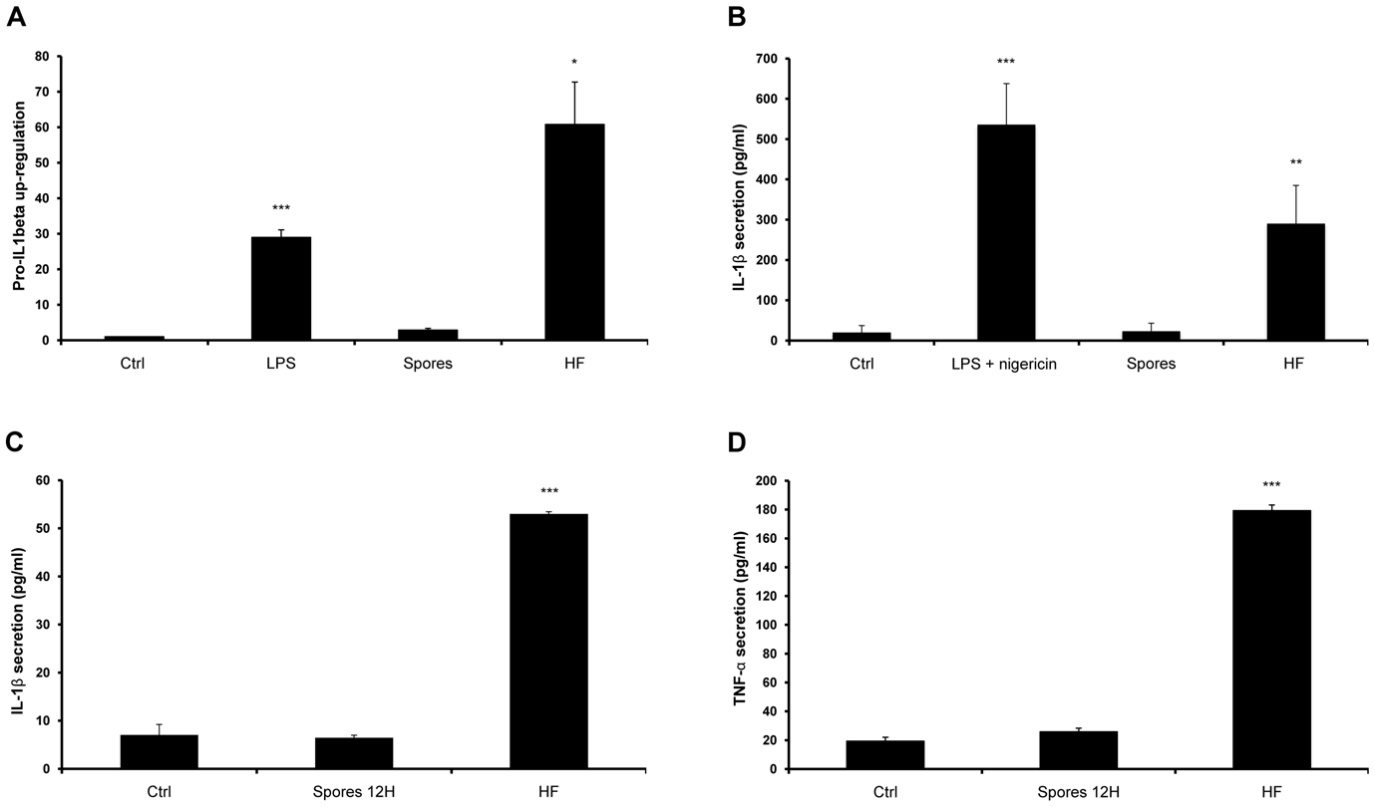
*A. fumigatus* hyphae upregulate pro-IL-1β transcription and induce IL-1β secretion in monocytes. One million THP-1 cells/ml were treated with 10 ng/ml of LPS with and without nigericin, spores or HFs at an MOI = 10 for 6 hours (A, B), or spores for 12 hours and HFs for 6 hours (C, D). (**A**) Intracellular IL-1β gene transcription was quantified by real-time PCR and compared to control. (**B, C**) The amount of secreted IL-1β was quantified by ELISA. (**D**) TNFα secretion was measured in supernatants by ELISA. Error bars represent standard deviation of at least three separate experiments. * *p*<0.05; ** *p*<0.01; *** *p*<0.001, compared to infected untreated cells.

To assess whether *A. fumigatus* could induce directly the synthesis or secretion of IL-1β, we examined the effect of different morphological forms of this fungus. The human monocyte cell line, THP-1, was infected with either conidia at a multiplicity of infection (MOI) of 10, or hyphal fragments (HFs) for 6 hours. As a positive control, the cells were primed with 10 ng/ml of lipopolysaccharide (LPS) for 6 hours in order to stimulate pro-IL-1β protein synthesis, with or without subsequent treatment with an NLRP3 inflammasome stimulator, the bacterial toxin nigericin for 1 hour. Real time PCR analysis showed that a 6 hour incubation with HFs induced a drastic increase in transcription of this pro-inflammatory cytokine gene, while spores provoked only a 2-fold increase (Figure 1A). Analysis of the supernatants by ELISA revealed that mature IL-1β was secreted from cells treated with LPS and nigericin, or only infected with HFs (Figure 1B). However, no significant secretion, beyond basal cytokine secretion levels, was seen when the cells were incubated with conidia (Figure 1B), consistent with the inability of this fungal form to induce transcription of the cytokine. To further investigate the potential of conidia to induce a pro-inflammatory cellular response, we incubated THP-1 cells in the presence of *Aspergillus* spores for a longer period of time, long enough to initiate the germination process. IL-1β and TNFα secretion was measured in the supernatants 12 hours after infection with spores, compared to 6 hour stimulation with HFs. Interestingly, swollen conidia were unable to induce any significant IL-1 β or TNFα secretion whereas the HFs caused noticeable monocyte activation (Figure 1C,D). Thus, we conclude that in contrast to HFs, *A. fumigatus* spores are unlikely to be critically involved in initiating IL-1β-dependent inflammatory responses. We decided therefore to perform the subsequent experiments only with the HFs of *A. fumigatus*.

### *A. fumigatus*-induced caspase-1 activation correlates with ROS production and K^+^ efflux

**Figure 2:**
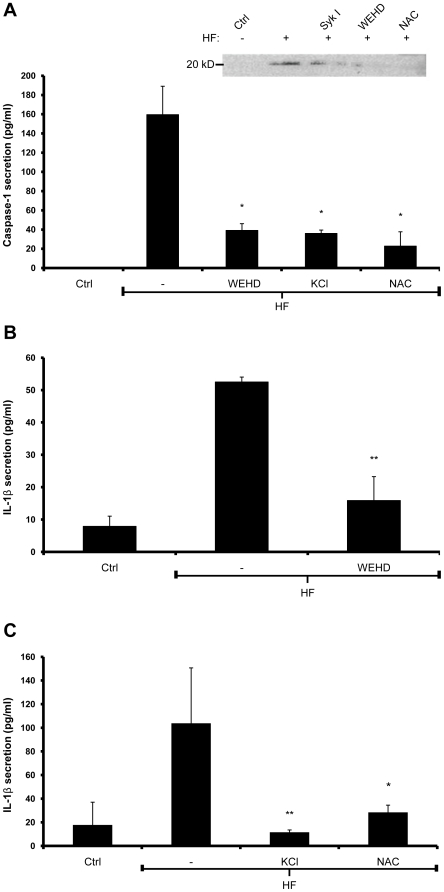
*A. fumigatus* induced-caspase-1 activation depends on ROS production and K^+^ efflux. THP-1 cells were incubated with HFs for 6 hours in the presence or absence of 130 mM KCl, 25 mM NAC, 100 µM caspase-1/caspase-5 inhibitor (Z-WEHD-FMK), or pretreated for 30 min with 1 µM of Syk kinase inhibitor (Syk I). (**A inset**) Caspase-1 activation was analyzed by Western blot, using an antibody against the Caspase-1 p20 cleavage product. Each band intensity was measured by NIH ImageJ software (Ctrl = 1, HF = 4.848, HF + Z-WEHD-FMK = 2.92, HF + Syk I = 1.67, and HF + NAC = 1.54). (**A**) Secreted Caspase-1 p20 and (**B, C**) mature IL-1β p17 in the supernatant of infected cells, compared to the control, was assessed by ELISA. Error bars represent the standard deviation of at least three separate experiments. * *p*<0.05; ** *p*<0.01; *** *p*<0.001, compared to infected untreated cells.

Caspase-1 activation is essential for pro-Il-1 β cleavage and subsequently IL-1β secretion. In fact, cells stimulated only with HFs activate caspase-1, as detected by Western Blot analysis of the cell lysates and supernatant by the appearance of the active p20 subunit of caspase-1 (Figure 2A, inset). This result was confirmed by measuring the presence of caspase-1 p20 subunits whose activated form is secreted into the supernatant of infected THP1 cells, as detected by ELISA (Figure 2A).

Caspase-1 activation is remarkably reduced in the presence of the irreversible caspase-1 inhibitor (Z-WEHD-FMK) (Figure 2A). Consistent with this result, Il-1β secretion induced by *Aspergillus* HFs was significantly decreased when monocytes were pretreated with Z-WEHD-FMK, again confirming the requirement for caspase-1 activation for Il-1 β secretion (Figure 2B).

A common feature of NLRP3 inflammasome activation by diverse stimuli is the cell-signaling pathway relying on K^+^ efflux and, concomitantly, production of reactive oxygen species (ROS) [34], [38]. To test the role of each of these variables, we first blocked K^+^ efflux by increasing the concentration of extracellular potassium, before stimulating the cells with HFs for 6 hours. IL-1 β secretion (Figure 2C) and caspase-1 activation (Figure 2A) were both significantly impaired by preventing K^+^ efflux. Comparable results were obtained when we used the antioxidant, N-acetyl-cysteine (NAC), as both IL-1β secretion and caspase-1 activation were strongly inhibited by incubating cells with NAC during exposure to *Aspergillus* HFs (Figure 2A,C). Thus, we conclude that ROS production and K^+^ efflux are essential for HF-induced caspase-1 activation and IL-1β secretion.

### The NLRP3 inflammasome in monocytes is stimulated by *A. fumigatus*

At least four inflammasomes have been described, based on: NLRP1 (Nalp1), NLRC4 (Ipaf), NLRP3 (Nalp3/cryopyrin), and AIM2 [34], [38], [39]. Murine Nalp1b detects anthrax LT, while NLRC4 recognizes mainly flagellin, and AIM2 is activated in response to cytosolic double-stranded RNA. Human Nalp1 is sensitive to peptidoglycan fragments [40]. Until now, the only inflammasome reported to be sensitive to cytosolic K^+^ concentrations and ROS contains NLRP3. We reasoned therefore that the NLRP3 inflammasome may be responsible for caspase-1 activation in response to *A. fumigatus* infection.

**Figure 3:**
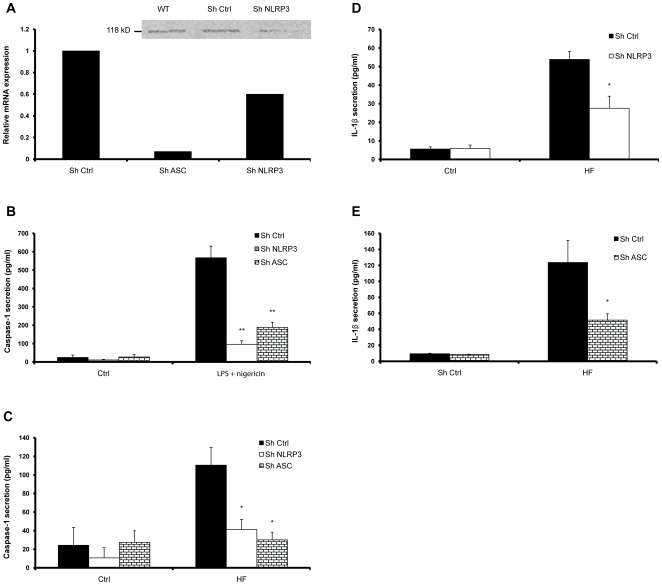
The NLRP3 inflammasome controls the anti-*A. fumigatus* innate immune response. THP-1 cells were stably transfected with shRNA targeting NLRP3 or ASC in order to induce gene silencing. (**A inset**) Western blot analysis of wildtype (WT) cells, cells treated with non-target control (SH control), and cells treated with shNLRP3, confirming decreased expression of the NLRP3 protein after mRNA depletion. Western blot was performed with an anti-NLRP3 antibody, which detects the 118 kDa protein. (**A**) mRNA levels of NLRP3 and ASC were quantified by real-time PCR and compared to wild type (WT) and non-target control (SH Ctrl). Supernatants of each of the knocked down (KD) cells treated with nigericin after LPS priming, or HFs for 6 hours was analyzed by ELISA for the presence (**B, C**) caspase-1 p20 and (**D, E**) mature IL-1β. All values are representative of at least three independent experiments. The error bars represent the standard deviation of at least three separate experiments. * *p*<0.05; ** *p*<0.01; *** *p*<0.001, compared to infected untreated cells.

The role of NLRP3 and its adaptor protein, ASC, was determined by gene silencing in THP1 cells. The mRNA expression levels of either inflammasome component was significantly reduced in knocked down (KD) cells, in comparison to non-target shRNA, as measured by real-time PCR (Figure 3A). Protein depletion was also confirmed using Western blot analysis (Figure 3A, inset). Because secretion of mature IL-1β after stimulation of primed THP-1 cells with nigericin relies primarily on NLRP3/ASC inflammasome activation, we examined IL-1β secretion in ASC KD and NLRP3 KD cells as a functional control. THP-1 KD cells secreted significantly less IL-β when stimulated with LPS and nigericin, demonstrating the efficiency of NLRP3 and ASC gene silencing (Figure 3B). In addition, these cells showed dramatic reductions in IL-1β secretion and caspase-1 activation in response to HFs, when compared to cells transfected with non-target shRNA (Figure 3C,D,E), which also correlated with the extent of mRNA depletion in the KD cells. The decrease in caspase-1 activation and IL-1β secretion in the KD cells implies that *A. fumigatus* infection induces caspase-1 activation through a process that requires, at least partially, the assembly of the NLRP3 inflammasome.

### Syk kinase provides both the first signal for IL-1β synthesis and the second signal for caspase-1 activation during *A. fumigatus* infection

**Figure 4:**
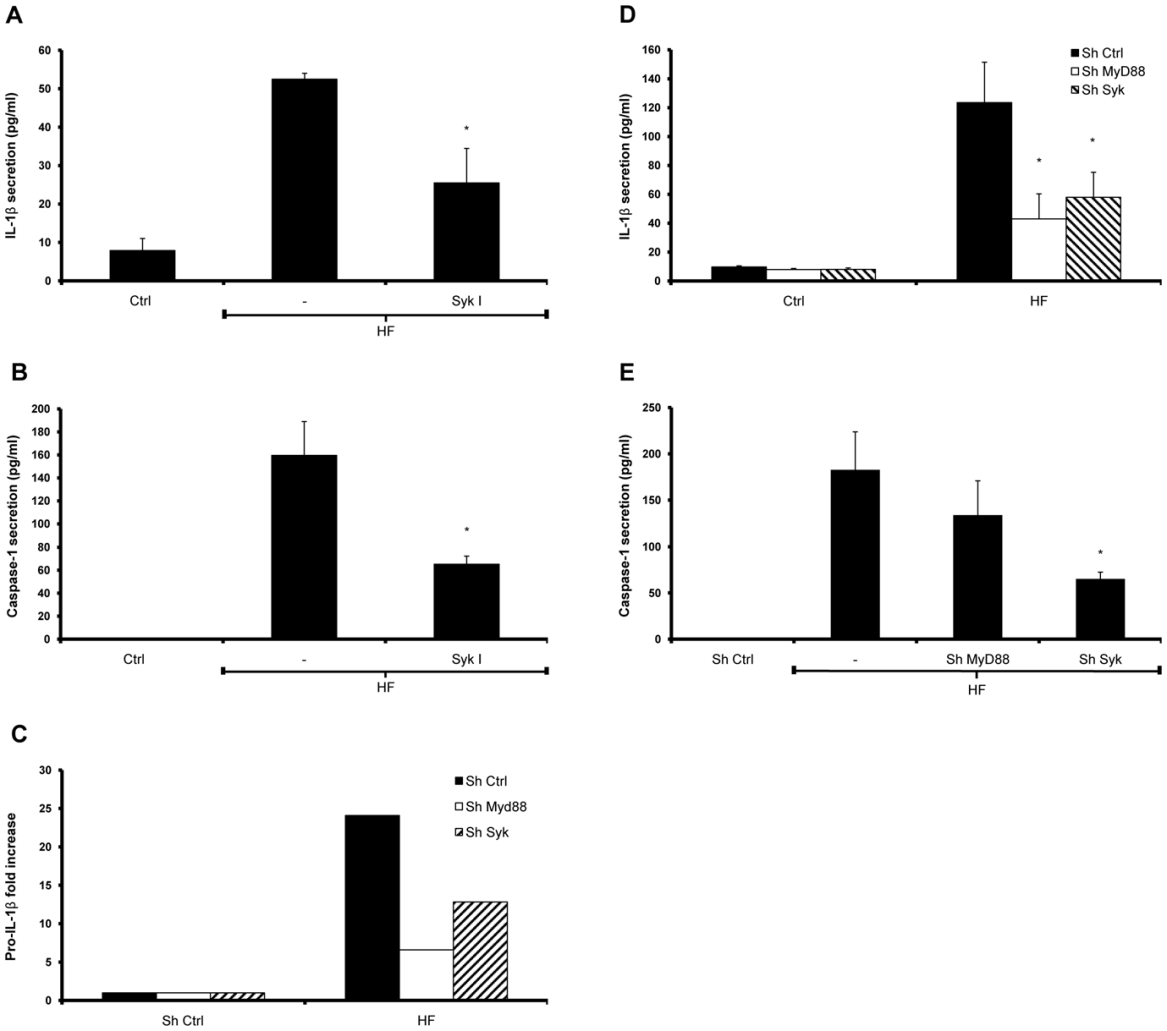
Syk kinase signaling provides the stimulus for both IL-1β synthesis and caspase-1 activation during *A. fumigatus* infection. THP-1 cells were pretreated with 1 µM of the Syk kinase inhibitor (Syk I) for 30 min prior to challenge with HFs, and (**A**) mature IL-1β and (**B**) active caspase-1 p20 subunit were measured by ELISA. MyD88 and Syk were stably silenced by RNA interference using shRNA. (**C**) Transcript levels of pro-IL-1β in MyD88 KD and Syk KD cells treated with HFs was measured using real-time PCR. Representative real-time PCR values representative of three independent experiments are shown. The secretion (**D**) of IL-1β and (**E**) caspase-1 p20 into the supernatants of MyD88 KD and Syk KD cells treated with HFs was assessed by ELISA. All values are representatives of at least three independent experiments. Error bars represent standard deviation of at least three separate experiments. * *p*<0.05; ** *p*<0.01; *** *p*<0.001, compared to infected untreated cells.

Previous studies have shown that *Aspergillus* hyphae and conidia have morphologically distinct and complex features that undergo several modifications during swelling. The composition of the conidial cell wall is complex and has not been completely defined, whereas hyphae contain mainly four major carbohydrate polymers of which one, the β-glucans, can activate dectin-1 in alveolar macrophages [41]. This receptor uses an intracellular ITAM motif to initiate signaling through a tyrosine kinase, Syk, in a MyD88-independent manner [42], [43]. Recent studies have revealed the importance of Syk in inducing NF-κB activation and controlling NLRP3-dependent caspase-1 activation during *C. albicans* infection. To examine whether Syk is involved in signaling during *A. fumigatus* infection, we blocked its signaling with a specific Syk inhibitor (Syk I) prior to HF exposure and measured IL-1β and caspase-1 secretion into the supernatant. The results suggest that Syk activation is indispensable for IL-1β secretion and caspase-1 activation (Figure 4A,B).

In order to further investigate the role of Syk, we used shRNAs to knock down separately Syk and MyD88. The adaptor protein MyD88 acts downstream of TLRs and is responsible for NF-κB activation. MyD88-specific gene silencing was confirmed in THP1 KD cells by real-time PCR (not shown). Since *A. fumigatus* stimulates TLR2/4 and Dectin-1 [12], knocking down Syk and MyD88 resulted in a large decrease in transcription of the gene encoding IL-1β, as shown by real time PCR (Figure 4C), and simultaneously an abrogation of IL-1β secretion by THP-1 cells (Figure 4D), compared to the SH control cells. However, only Syk KD cells presented a significant reduction in caspase-1 activation when stimulated with HFs for 6 hours (Figure 4E). These results imply that signaling through Syk and MyD88 both converge on NF-κB activation during innate responses against *A. fumigatus* infection, but only Syk signaling results in NLRP3 inflammasome activation.

### *A. fumigatus* PAMP recognition is impaired in the presence of corticosteroids

**Figure 5:**
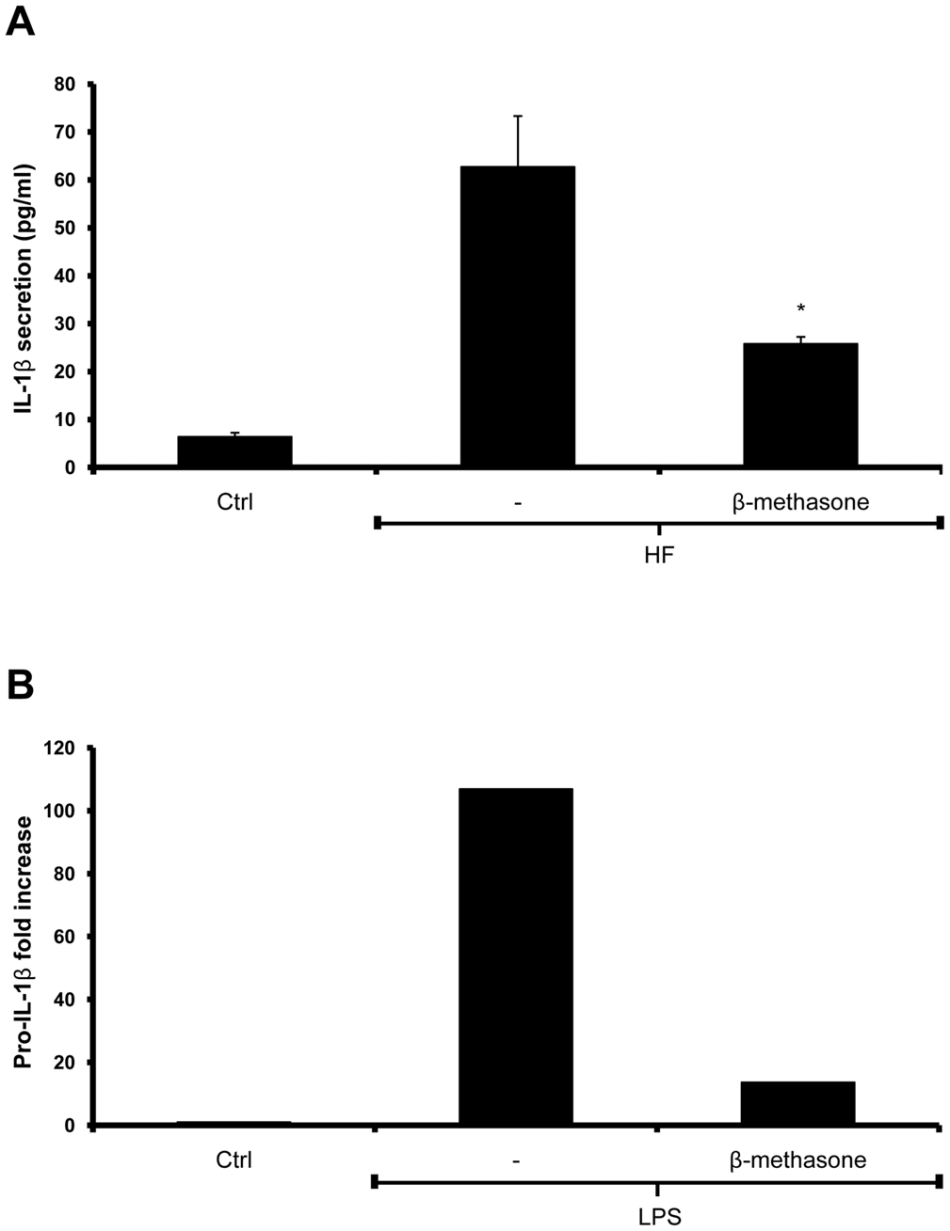
The inflammatory response against *A. fumigatus* is impaired in immunosuppressed monocytes. (**A**) THP-1 cells were stimulated for 10 min with 30 µM β-methasone prior to stimulation with HFs for 6 hours. IL-1β secretion was measured by ELISA. (**B**) THP-1 cells were stimulated for 10 min with 30 µM β-methasone prior to stimulation with 10 ng/ml LPS for 6 hours. IL-1β mRNA was quantified by real-time PCR.

In a large cohort study, *A. fumigatus*-infected patients who were under corticosteroid treatment were found to be at increased risk of subsequent invasive aspergillosis, suggesting a deleterious effect of these compounds on host anti-fungal resistance [44]. Therefore, it was of interest to determine whether corticosteroid treatment affects the ability of *A. fumigatus* to trigger secretion of IL-1β. Pre-incubation of THP-1 cells with β-methasone, a potent anti-inflammatory corticosteroid, followed by infection with HFs resulted in a significant drop in IL-1β secretion (Figure 5A). To distinguish between the ability of β-methasone to interfere with pro-IL-1β gene expression or caspase-1 activation, we observed that pro-IL-1β transcription induced by LPS is severely defective when THP-1 cells were pretreated with β-methasone (Figure 5B). These results show that corticosteroids inhibit primarily the ability of monocytes to transcribe the gene for IL-1β, and may partially explain why patients treated with corticosteroids fail to produce pro-inflammatory cytokines, which are crucial for recruitment of other immune cells to clear infections.

## Discussion

Stimulation of PRRs (TLR-2, TLR-4, and dectin-1) during infection with *A. fumigatus* leads to activation of transcription factors such as NF-κB, whose translocation into the nucleus stimulates upregulation of pro-inflammatory cytokines. In contrast, inflammasome assembly during aspergillosis has never been described, although previous studies showing secretion of IL-1β by THP-1 during infection by *A. fumigatus* [19] suggest that an inflammasome and caspase-1 must be activated. Our studies reveal that *A. fumigatus* does in fact stimulate both pro-IL-1β production and caspase-1 activation, leading to mature IL-1β secretion into the supernatant. However, our results suggest that only *Aspergillus* hyphae, and not conidia, are capable of inducing inflammasome assembly and caspase-1 activation in monocytes. Furthermore, we showed that a NLRP3 inflammasome is involved in caspase-1 activation, since there was a profound suppression of IL-1β release from NLRP3 and ASC knocked-down cells. We also showed that the adaptor protein, ASC, is required for inflammasome activity. The list of NLRP3 inflammasome activators is growing, but the mechanisms by which this NLR family member senses its activators seem to converge on a small number of intracellular perturbations such as K^+^ efflux and ROS production [38]. Our data confirmed that *A. fumigatus*-induced NLRP3 inflammasome activation in monocytes is associated with K^+^ efflux and ROS production, since their inhibition resulted in a significant decrease of caspase-1 activation and IL-1β secretion.

Since *A. fumigatus* expresses ligands for several PRRs, it is likely that these ligands cooperate in transducing diverse signals. Our studies with HFs are consistent with synergy between TLR-2, TLR-4 and dectin-1 signaling, since depletion of MyD88 and Syk significantly reduced pro-IL-1β production. Moreover, our results highlighted the role of the Syk kinase as an inflammasome activator in *Aspergillus* infection, and in contrast, ruled out any involvement of MyD88 signaling in caspase-1 activation.

Disease caused by *A. fumigatus*, which is mostly nonpathogenic for humans, is closely associated with the status of the host immune system, particularly the innate immune system, rather than the pathogenicity of the fungal pathogen. In fact, immunodeficiency is a primary factor predisposing patients and animals to severe IA. Here, we show that treatment with the corticosteroid, beta-methasone, which induces immunosuppression, translated into failure of human monocytes to produce IL-1β in response to LPS or *Aspergillus* hyphae. Pro-inflammatory cytokines are crucial for stimulating an effective immune response to *A. fumigatus* infection, which includes recruitment of neutrophils to the alveolar spaces, where they constitute more than 90% of the phagocytic cells [12].

Taken together, our data thus demonstrate that the innate immune response against *A. fumigatus* infection involves a two step activation process, with a first signal, due to TLR and dectin-1 ligation, promoting expression and synthesis of pro-IL1β; and a second signal, involving Syk-induced activation of the NLRP3 inflammasome and caspase-1. Both signals, together, allow secretion of mature IL-1β. In many immunosuppressed patients, susceptibility to *A. fumigatus* infection could be caused by failure to provide an effective response to the first signal.

## Materials and Methods

### *Aspergillus* growth and culture

*A. fumigatus* strain AFCOH1, isolated from patients at the City of Hope National Medical Center (Duarte, CA) and kindly provided by Drs. Joseph Lyons and Markus Kalkum (City of Hope), was grown 5 to 7 days at 37°C in potato dextrose agar (BD/Difco). Conidia were extracted from agar slants by gentle tapping and resuspended into PBS containing 0.1% Tween 80 (PBS/Tw). Clumps of conidia were dispersed with 3 mm glass beads, washed with PBS/Tw and suspended in 30% glycerol. Aliquots were frozen at −80°C and thawed to 37°C prior to use as described previously [45].

To induce hyphal growth, 10^7^ spores/ml were inoculated in 50 ml of potato broth (BD/Difco) and incubated for 24 hours under 200 rpm agitation at 37°C. The mycelium was then dried down onto Whatmann 54 paper using a Buckner funnel and a side-arm flask attached to a vacuum pump. Hyphae were washed 3 times with 0.6 M MgSO_4_, and resuspended in PBS/Tw. To yield hyphal fragments (HFs), this mycelium suspension was broken down under vigorous vortexing in the presence of 3 mm glass beads and stored at 4°C for up to one week.

### Reagents and cell line

The human acute monocytic leukemia cell line (THP-1) was obtained from American Type Culture Collection (ATCC). N-acetyl cysteine (NAC), glibenclamide, beta-methasone and *Echerichia coli* LPS were from Sigma (St. Louis, MO). KCl was from Fisher Scientific, Syk inhibitor was from Calbiochem (Cat. No. 574711) and Z-WEHD-FMK was purchased from R&D Systems (Minneapolis, MN).

### Cell culture infection and treatments

THP-1 cells were cultured in tissue culture flasks (Costar, Corning, NY) using RPMI medium (Gibco) supplemented with 10% heat-inactivated fetal calf serum (Gibco) and incubated in a humidified incubator at 37°C with 5% CO_2_. One million cells/ml were plated in medium and conidia or HFs were added at a multiplicity of infection (MOI) of 10 and incubated for 6 hours at 37°C with 5% CO_2_. Cells were spun down at 1200 rpm, 4°C for 5 min, and supernatants were stored at −80°C for cytokine assay use and pellets are resuspended in the appropriate lysis buffer for RNA extraction or Western blot analysis. Treatment with inhibitors or other reagents was performed at the indicated times and concentrations.

### Generation of THP-1 cells expressing shRNA

THP-1 cells stably expressing shRNA against NLRP3 and ASC were obtained by transducing THP-1 cells with lentiviral particles. The sequences 5′- CCGGGCGTTAGAAACACTTCAAGAACTCGAGTTCTTGAAGTGTTTCTAACGCTTTTTG-3′ for human NLRP3 (Sigma; Cat. No. NM_004895), 5′-CCGGCGGAAGCTCTTCAGTTTCACACTCGAGTGTGAAACTGAAGAGCTTCCG TTTTTG-3′ for human ASC (Sigma; Cat. No. NM_013258), 5′-CCGGCCTGTCTCTGTTCTTGAACGTCTCGAGACGTTCAAGAACAGAGACAGGTTTTT-3′for human MyD88 (Sigma; Cat No. NM_002468), and 5′-CCGGGCAGGCCATCATCAGTCAGAACTCGAGTTCTGACTGATGATGGCCTGCTTTTT-3′ for human spleen tyrosine kinase (Syk) (Sigma; Cat #: NM_003177) were used separately to silence gene expression following the manufacturer's instructions. Non-target shRNA control cells were also generated using an irrelevant sequence (Sigma; Cat. No. SHC002V).

### Western blotting

Samples were lysed using RIPA Lysis Buffer (Millipore) and loaded onto a 15% SDS-PAGE gel, and then transferred to a polyvinylidene difluoride membrane (Millipore) as we previously described [46]. Blots were blocked for 1 hr with 5% (w/v) nonfat dried milk in TBST. The membrane was incubated overnight at 4°C with rabbit antihuman caspase-1 antibody (Millipore) followed by an incubation with a conjugated anti-rabbit IgG horseradish peroxidase (Millipore). For confirmation of NLRP3 depletion by RNA interference, a 9% gel was used and the blot was incubated with rabbit anti-human NLRP3 antibody (Sigma; Cat. No. HPA012878). Immunoreactive proteins were detected with ECL Plus Western Blotting Detection Reagents (Amersham, Scituate, MA) using a gel doc system (Biorad, Hercules, CA). Intensity of bands was determined using NIH ImageJ software [47].

### RNA isolation and real-time PCR

mRNA was isolated from THP-1 cells using the Qiagen RNeasy kit (Qiagen, Valencia, CA) following manufacturer's instructions, and total RNA was converted into cDNA by standard reverse transcription with Taqman® reverse transcriptase kit (Applied Biosystems, Foster City, CA). Quantitative PCR was performed with 1/50 of the cDNA preparation in an Mx3000P (Stratagene, La Jolla, CA) in a 25 µl final volume with Brilliant QPCR Master Mix (Stratagene). The primers for human GAPDH were: 5′- CTTCTCTGATGAGGCCCAAG-3′ forward, 5′GCAGCAAACTGGAAAGGAAG-3′ reverse. Primers for human NLRP3: 5′- CTTCCTTTCCAGTTTGCTGC-3′ forward, 5′-TCTCGCAGTCCACTTCCTTT-3′ reverse. Primers for human ASC: 5′-AGTTTCACACCAGCCTGGAA-3′ forward, 5′- TTTTCAAGCTGGCTTTTCGT-3′ reverse. Primers for Syk: 5′-AGAGCGAGGAGGAGCGGGTG-3′ forward, 5′-CCGCTGACCAAGTCGCAGGA-3′ reverse. Primers for MyD88: 5′AGCGCTGGCAGACAATGCGA-3′ forward, 5′-TCCGGCGGCACCTCTTTTCG-3′ reverse. Primers for Il-1β: 5′-CAGCCAATCTTCATTGCTCA-3′ forward, 5′-TCGGAGATTCGTAGCTGGAT-3′ reverse. The real-time PCR included an initial denaturation at 95°C for 10 min, followed by 40 cycle of 95°C for 30 s, 55°C for 1 min, 72°C for 1 min, and one cycle of 95°C for 1 min, 55°C for 30 s, 95°C for 30 s.

### ELISA measurement of cytokine and caspase

Commercially available ELISA kits for human IL-1β (Ebioscience) and human caspase-1 (R&D systems) were used according to the manufacturers' instructions.

### Statistical analysis

The difference between groups was performed using GraphPad Instat software (GraphPad Software Inc, La Jolla, CA) by Student's test. The level of significance between groups was set at *P *<0.05. All experiments were performed at least 3 times (unless stated otherwise) and the data was presented as the cumulative result of all the experiments done.
